# MEFV and NLRP3 gene variants in children with pfapa syndrome in slovenia

**DOI:** 10.1186/1546-0096-12-S1-P81

**Published:** 2014-09-17

**Authors:** Daša Perko, Maruša Debeljak, Nataša Toplak, Tadej Avčin

**Affiliations:** 1 Department of Allergology, Rheumatology and Clinical immunology, UMC Ljubljana, University Children's Hospital Ljubljana, Slovenia; 2 Unit for Special Laboratory Diagnostics, UMC Ljubljana, University Children's Hospital, Ljubljana, Slovenia

## Introduction

PFAPA syndrome is the most common autoinflammatory fever disorder in childhood, characterized by recurrent fever, aphthous stomatitis, pharyngitis and adenitis. Mutations in the *MEFV* and *NLRP3* genes are known to cause syndromes with PFAPA overlapping symptoms (Familial Mediterranean Fever and Cryopyrin-Associated Periodic Syndrome), which are rarely reported in patients from Slovenia.

## Objectives

The aim of the study was to assess the frequency of *MEFV* and *NLRP3* gene variants in pediatric patients with PFAPA syndrome from Slovenia in order to determine whether genes involved in other autoinflammatory diseases, might play a role in PFAPA pathogenesis.

## Methods

We collected clinical and laboratory data of PFAPA patients under the age of 5, who were followed at the University Children's Hospital Ljubljana. All 10 exons of *MEFV* gene and 9 exons of *NLRP3* gene, including intron/exon regions of both genes were directly sequenced.

## Results

In total, 30 PFAPA patients were tested for *MEFV* and *NLRP3* gene variants. Mean age at the syndrome onset was 2.1±1.3 and at diagnosis 4.2±1.8 years. 19(63%) patients were male and 11(37%) were female. Mean duration of episode was 3.5 days, mean interval between the episodes was 3.5 weeks. Most common symptoms beside fever were pharyngitis and cervical adenitis in 90% and aphtosis ( always or sometimes) in 63%.

Overall, 10 patients (33%) were found to have 11 variants, all in heterozygous state. 6 patients have Q703K variant in *NLRP3*, one E148Q in *MEFV* and one combination of I591T in *MEFV* and Q703K in *NLRP3*. Novel variant in *NLRP3*, P200T, was identified in one patient. One girl was found to have known variant in *NLRP3* gene, S726G, which is associated with CINCA syndrome. This girl has had typical PFAPA symptoms, but she also has epilepsy and mild developmental delay.

## Conclusion

Five different *MEFV* and *NLRP3* gene variants were identified in 10 of 30 PFAPA patients with *MEFV* variants found in 2 patients and *NLRP3* variants in 9. Our results indicate genetic heterogeneity of PFAPA population and possible overlap with other periodic fever syndromes.

## Disclosure of interest

None declared.

